# A Service Discovery Solution for Edge Choreography-Based Distributed Embedded Systems

**DOI:** 10.3390/s21020672

**Published:** 2021-01-19

**Authors:** Sara Blanc, José-Luis Bayo-Montón, Senén Palanca-Barrio, Néstor X. Arreaga-Alvarado

**Affiliations:** 1 Institute of Information and Communication Technologies, ITACA, Universitat Politècnica de València, Camino de Vera s/n, 46022 Valencia, Spain; jobamon@itaca.upv.es (J.-L.B.-M.); nesaral@upv.es (N.X.A.-A.); 2 School of Informatics, ETSINF, Universitat Politècnica de València, Camino de Vera s/n, 46022 Valencia, Spain; sepabar@inf.upv.es; 3 Escuela Superior Politécnica del Litoral, ESPOL, Polytechnic University, FIEC, Campus Gustavo Galindo Km. 30.5 Vía Perimetral, P.O. Box, Guayaquil 09-01-5863, Ecuador

**Keywords:** Internet of Things, service choreography, middleware, distributed embedded systems, edge, fog computing, discovery, regular expressions

## Abstract

This paper presents a solution to support service discovery for edge choreography based distributed embedded systems. The Internet of Things (IoT) edge architectural layer is composed of Raspberry Pi machines. Each machine hosts different services organized based on the choreography collaborative paradigm. The solution adds to the choreography middleware three messages passing models to be coherent and compatible with current IoT messaging protocols. It is aimed to support blind hot plugging of new machines and help with service load balance. The discovery mechanism is implemented as a broker service and supports regular expressions (Regex) in message scope to discern both publishing patterns offered by data providers and client services necessities. Results compare Control Process Unit (CPU) usage in a request–response and datacentric configuration and analyze both regex interpreter latency times compared with a traditional message structure as well as its impact on CPU and memory consumption.

## 1. Introduction

The latest innovations in technology and communication allow for flexible adaptation of the Internet of Things (IoT) paradigm to many application areas. A layered architecture benefits the adaptation. We can find several examples that propose interconnected layers from the sensors to the end user [1,2,3,4,5]. The complexity of these layers depends on the application objectives and magnitude of the observation, the ubiquity of sensors and actuators, and the available infrastructure.

Many scenarios could benefit from the advantages of an IoT architecture which includes an edge or fog computing layer able to carry out data storage, control management, decision making, service integration, and intra- and interoperability.

However, building an edge computing layer with embedded devices is a challenge. The computational capacity of machines is usually between the low capacity of the sensors and the high performance of cloud systems.

In advanced edge systems, machines need to exchange information to make decisions. Orchestration and choreography models describe how this information should flow [6,7]. On the one hand, orchestration fits into a centralized model for service connection where a central element manages the state of the process and the flow of data [8]. On the other hand, choreography relies on a combination of distributed services that cooperate to provide functionality and manage the processes and the flow of data. This makes choreography appropriate to apply to distributed edge services with distributed data storage in machines with similar computing capabilities.

Choreography in embedded systems specifies interactions among services [9,10]. Every service collaborates to provide functionality. Choreographies support mechanisms for a highly coupled integration of services, as compared to the loosely coupled integration promoted in traditional service oriented architecture (SOA) systems.

In this paper, choreography is supported by the message passing engine in [11] which allows for routing service invocations, as the lowest layer of a middleware that is implemented on top of the transport layer. The engine efficiently exploits performance and use of system resources, making it suitable for building edge devices oriented to the IoT [12].

Furthermore, the choreographer facilitates the growth and extension of the ICT system, providing a complete integration platform [13]. New, heterogeneous services can be easily incorporated, removed, substituted, or moved to other choreographers in the system.

However, the engine does not include a discovery mechanism. Such a mechanism is necessary to allow dynamic data flow composition for blinded hot plugging of new machines. When a new machine is plugged into the system, services allocated in the machine do not know a-priori the identification of any network service with which it could connect to exchange data.

This paper proposes the implementation of a discovery service under this choreography message passing engine. The engine is deployed in several Raspberry Pi machines. Any machine with a choreograph engine is inside the choreography system. Each machine hosts a set of microservices interconnected by the engines transparently to their physical ubication and the transport layer.

We face two issues on building distributed embedded systems under a choreography-based message exchange model. On the one hand, the specific application scope, cyber‒physical deployment, and infrastructure of each possible edge scenario require flexible systems that support adaptable data exchange patterns. Specifically, our solution supports three models: request‒response, datacentric, and rules‒server. The three models coexist.

On the other hand, the need of reducing structural complexity of queries in the discovery process [14]. Regular expression has not been explored yet in embedded systems to support service discovery. Our solution uses regular expression (Regex) to discern both publishing patterns offered by a data provider and client necessities. It favors decisions to balance the load by selecting suitable associations between services.

The paper is organized as follows. Section 2 motivates the research work. Section 3 explains the proposed solution. Section 4 analyzes the results obtained in a testing prototype. Finally, Section 5 discusses the overall approach of this paper and how useful it could be in future development in IoT. Some open issues are provided too. Section 6 concludes the paper.

## 2. Materials and Methods

Service composition in choreographies describes how services can interact with each other at message level [15]. Choreography is applicable to edge and fog IoT layers, but implicit constraints of devices and machines at the edge layer make challenges arise.

The edge layer supports simple IoT devices that cannot undertake complex computational processes or that need data streams from several devices to execute an action. The offloading of data generated by sensors varies from improving the user experience by reducing latencies in availability [16], to making decisions in complex event processing systems (CEP) [17], among others.

The authors in [16] do not use choreography modeling but propose Data as a Service (DaaS) at the fog layer in [18]. Among data movement actions it includes to move or duplicate data from edge-to-edge storages. The work is focused on data quality and how a dataset can be considered useful for one application and inadequate for another application. Data is collected over long periods, thus not requiring real-time analysis. Although data quality is very relevant, we focus on making decisions in real time.

Lan et al. [17] propose an architecture with an IoT gateway or a separate computer in the same LAN as part of the edge layer. The gateway receives data from different sources and processes it with complex rules to provide complex events to cloud consumers.

We propose a similar organization and approach in the use of data. CEP concept could be applied on choreography, assuming consumers also at the edge level to generate automatic actions, as we propose in this paper.

In recent years, service composition modeling under choreography paradigm has been addressed in several platforms such as CHOReOS [19,20] and its evolution into CHOReVOLUTION [21]; ActnConnect [22]; ChorSystem [23]; and research works such as [24,25,26,27,28]. Extensions integrated within choreography add functionality oriented to different scenarios [29,30,31,32].

Usually, choreography models couple both control and data flow within the system workflow. To decouple data flow and control flow modeling, TraDE [29] is a middleware which supports cross-partners data flows by using data objects and intermediate storage. The choreography model applies TraDE modeling extensions to replace the traditional message-based data exchange by a data-aware choreography [30]. Previous works of TraDe authors include the ChorSystem platform [23].

In TraDE, a data object has a unique identifier which describes the type of data contained. Data objects are exposed in a web-accessible manner through a REST API.

TraDE efforts are mainly focused on efficiently placing and providing data. As an advantage, TraDE middleware keeps a copy of the data generated or transformed by service providers to be accessed at any time. A consumer will be able to locate and access the data through the middleware. Therefore, the dataflow is highly decoupled from data exchange. However, at the edge level, with an architectural approach like the one presented in [17], data storage tends to be ephemeral. Data generated by sensors are useful during a short period of time for immediate decision making. Over time, data is useful for machine learning or historical observation, among others. Therefore, it is sensible to store copies in the cloud. However, TraDe is an inspiration for us in organizing middleware integrated with choreography modeling.

Thus, focused on the edge level, and assuming the ephemeral use of data in automatic decision making, our approach integrates a middleware on choreography message exchange. The control flow and the association of the relationships between the modeling participants is decoupled. The objective is to offer the consumer different provided data options but without permanent data storage.

The work presented in [31] uses a choreography model to associate consumers with different providers. The approach is focused on dynamic configuration. For example, it is possible to change the association between lights and switches in a room without modifying the installed equipment and electrification. However, the system does not include data from sensors or the run-time plugging of new services.

Finally, the work in [32] is integrated within CHOReVOLUTION to support real-time information in traffic routing. The approach is promising in cooperative intelligent transport systems (C-ITS) where vehicles, infrastructure, and numerous cloud services are connected and cooperate for efficient transport solutions. Real-time service discovery is not approached but it could be compatible with the Chen et al. proposal.

The choreography message passing engine in [11] is light enough to be supported by typical machines at edge level as Raspberry Pi. The implementation was inspired by OASIS Web Services standards, and it has been tested and recently applied to previous IoT works [12]. The engine was created using the recommendations from the Foundation for intelligent Physical Agents (FIPA) [33]. Message headers typing is extracted from the Simple Object Access Protocol (SOAP) [34,35]. In case of needing to serialize the messages, both XML and JSON are supported, but also extensions.

Choreography at edge level aims to distribute computational load among machines. Edge machines can host multiple services. These services are independent but collaborate both inside and outside of the host to manage the wireless sensor networks (WSN), to process data, to make decisions, and to implement interoperability with external services.

**Figure 1:**
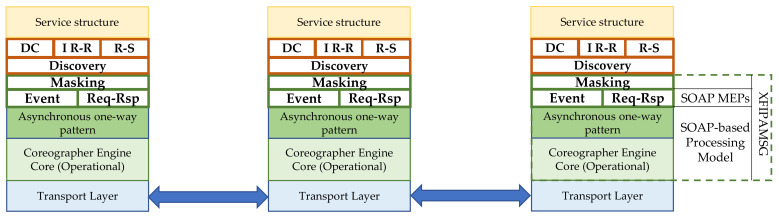
A layered structured supported by a choreograph engine.

In Figure 1, each machine hosts a message passing choreograph engine. In choreography, services are location-agnostic of other services. Thus, every sent message is forwarded to every choreographer in the system. Messages have a routing identifier. A service receives the message when the message identifier matches with one in its reception list. Moreover, the matching accepts AND and OR masking (Masking). Routing invocations through the choreography engine makes the location of services ubiquitous and the transport layer is transparent to services.

Regarding IoT data exchange, IoT messaging protocols define different communication patterns that are especially relevant to sensor data exchange [36]. For example, the Constrained Application Protocol (CoAP) [37] is a web transfer protocol based on the Hypertext Transfer Protocol (HTTP) that supports the request–response communication pattern. The Message Queuing Telemetry Transport Protocol (MQTT) [38] and MQTT-SN for Sensor Networks [39] support topic request–response. The Advanced Message Queuing Protocol (AMQP) [40] supports both request–response and topic-based publish-subscribe messaging. The Extensible Messaging and Presence Protocol (XMPP) standard supports request–response, asynchronous messaging and publish-subscribe [41]. Finally, the Data Distribution Service (DDS) [42] supports datacentric communication. The DataCentric Publish-Subscribe protocol (DCPS) of DDS delivers information to the receivers not based on their destination but rather as a function of the type of payload. Thus, datacentric pattern is a one-to-many communication model.

The choreograph engine in Figure 1 supports both immediate request–response (Req-Rsp) and asynchronous event messaging (Event) based on SOAP. The engine is built in a layered structure style which accepts new layers for new exchange patterns and protocols. Thus, our proposal adds datacentric (DC) and a type of publish-subscribe for rules (R-S) in addition to immediate request–response (IR-R).

**Figure 2:**
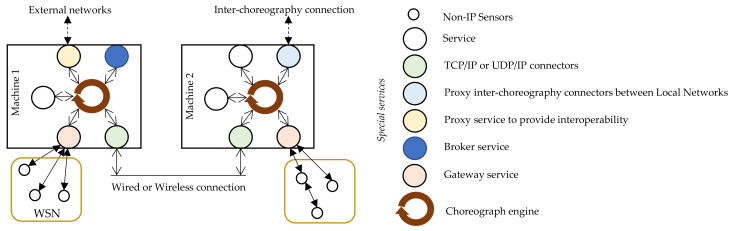
Choreography system at the edge level.

Figure 2 shows the service system. Gateway services receive data from a finite number of sensors, sometimes deep constrained sensors without the standard IP layer, that are deployed in a wireless sensor network (WSN) topology [43,44]. Many communication technologies are useful to build long-range and low-power wireless communication [45,46]. Thus, the distance between sensors and gateways is no longer a problem.

Gateway services receive data which are stored in local databases to each machine. Data are processed or distributed to other services in the edge within a communication pattern.

Regarding service discovery, dynamic discovery has been implemented as a specific service called broker which manages discovery and subscription updates while messaging is carried out without broker intervention. Blind hot plugging allows the broker to balance the load by selecting associations between services. For example, if a client needs temperature data in an area where there are two or more sensors, the broker selects the one that is least used for request–response, or the one that is already sending data using datacentric pattern.

Finally, in Figure 2, the choreography system includes proxies as components to provide interoperability with other networks. A similar concept has been applied to other works as in [47].

To facilitate interoperability, the discovery mechanism is inspired by the Web Services Dynamic Discovery (WS-Discovery) OASIS standard protocol [48]. However, as mentioned in [49] or [50], implementing WS standards directly on embedded devices is not always straightforward.

Recent works in IoT discovery techniques appeal to the use of data semantic description. For example, in [51] the authors introduce a data stream centric ontology and in [52] discuss the need of semantic data annotation in message syntax at the IoT edge level in gateways to facilitate interoperability but considering machine constraints. However, the description of message information by using structured formats generates long messages.

Typical constraints in embedded devices suggest the necessity of providing lightened structures that are still compatible with standards. Using regular expressions could reduce the size of messages.

Eventually, to interconnect all these services, any service needs both to support a list of message identifiers that as receiver accepts with or without mask and a dynamic mechanism to discover new identifiers to be added to its receiver list. Both mechanisms are explained in the next section.

## 3. Basic Concepts

Services are implemented on top of the choreograph engine and are organized following two principles:

1. Incoming data from sensors are stored among all interconnected edge machines in a distributed way. Assuming an edge computing layer composed by storage limited devices, this principle maximizes the use of memory.
2. Discovery is managed by a unique broker server in the choreography system. This principle maintains consistency and facilitates the efficient use of service providers.

In a data distributed system, where data are stored among small memory units, packet exchange is mainly focused on the exchange of data. Each service can be a provider or a consumer, or both (prosumer).

- Data providers. Receive data from sensors and deliver data to other services.
- Data consumers. Receive data from other services and maintain interoperability.

**Figure 3:**
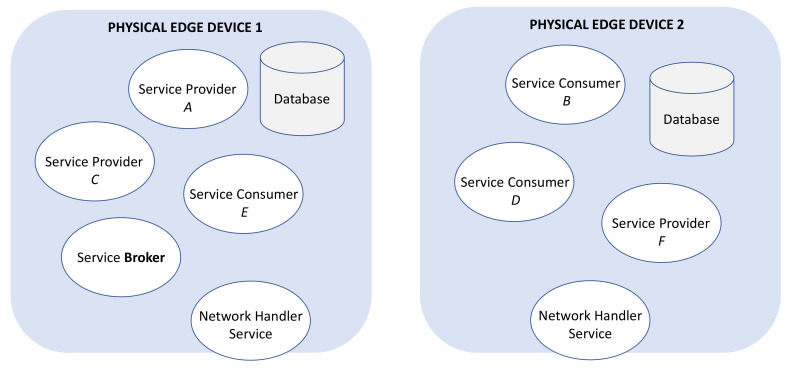
Physical service level.

**Figure 4:**
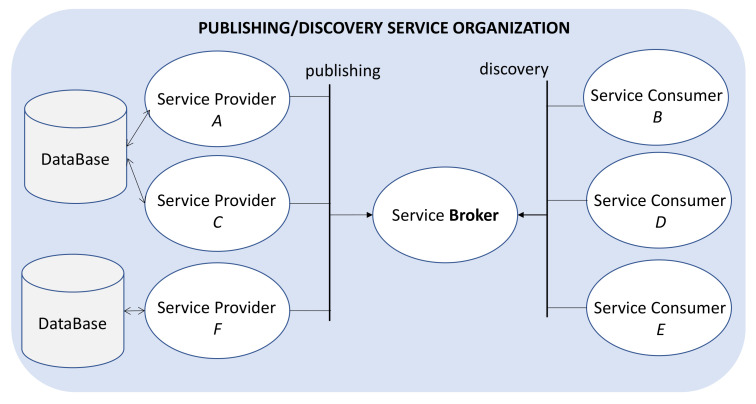
Logical service abstraction level.

For example, Figure 3 shows in blue two physical edge devices that contain a database and several service providers and consumers. The logical abstraction layer inside the choreography system is shown in Figure 4.

Each machine adds specific TCP/IP or UDP/IP service connectors to handle network and transport layers to interconnect the physical devices.

**Figure 5:**
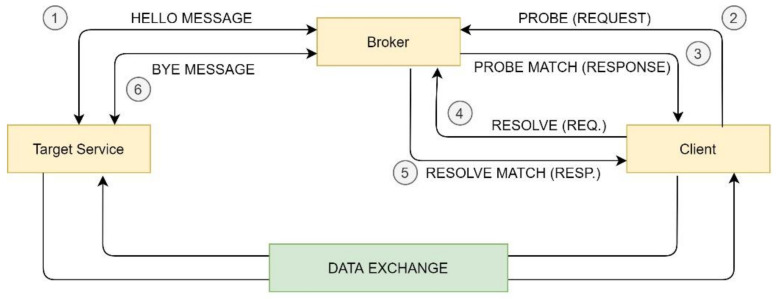
Web Service Discovery: a general view.

Figure 5 shows a general view of discovery based on WS-Discovery. HELLO and BYE messages are part of the publishing mechanism, while PROBE, PROBE MATCH, RESOLVE, and RESOLVE MATCH are part of the discovery protocol. The following subsections analyze these mechanisms.

### 3.1. Discovery

To allow blind hot plugging, consumers need a flexible discovery mechanism. The broker manages both the publication and discovery processes. To facilitate an interoperability layer with external web services, the broker implements a lightweight packets format based on WS-Discovery. Target services are service providers and clients are consumer services.

To be compatible with services outside of the local area network (LAN), a proxy could easily transform WS-* native messages into choreography WS-Discovery messages. WS headers have been adapted to choreography message headers. Regarding the body of the message, it explicitly includes the sensors and measurements, but the broker interprets the subscription pattern with the Regex class [53]. The interpretation of natural language with regular expressions facilitates the integration of heterogeneous services and a blind hot plugging where the consumer does not know in advance the service or services with which they can connect. Interpretation is based on the discernment of the delivery pattern, the delivery frequency in case of events, and rules applicable to the rule‒server pattern. The Regex approach serves the broker by interpreting information contained in the “scope” of the body. Some examples are given in Section 4.

Client services carry out the discovery process by incorporating regular expressions into the message scope. As a client could accept one or several delivery patterns, to express such a diversity only with basic expressions, the scope would be built with a nested structure. However, the use of regular expressions allows one to express the diversity in a single scope. Thus, depending on the current network traffic, the broker tries to balance the use of the patterns, prioritizing datacentric. Thus, interpretation also implies traffic balance.

### 3.2. Communication Patterns

Each new provider that plugs into the system publishes its “provision” to the broker service. The provision contains metadata and a body. The broker maintains a dynamic list that is updated with each new publication. Metadata caters to the service address identifier and the body includes the type of sensor and measurement, gathering frequency and accepted data delivery patterns.

Service providers in Figure 4 are published through the broker, attending to three possible data delivery patterns: request‒response, datacentric, and rules‒server. These patterns indicate how the provider can deliver data to consumers. Following the WS-Discovery, each new consumer plugged into the system asks the broker about a better possible match.

The broker acts as the service network manager and checks if there is any provider that meets the requested requirements and resolves the request with either a null, no provision, or with the identifier of the provider. Both publishing and subscription must be coincidental in the adopted pattern.

#### 3.2.1. Request‒Response Pattern

Request‒response subscription is at least one quality of service (QoS) pattern initialized by a service that requires a very specific answer from another service. A service provider published in this mode attends to requests that are specifically addressed to it.

Suppose we have provider A and consumer B, as in Figure 4. B needs to request a dataset from A directly, e.g., the ambient measurements recorded in the database over the last hour. Thus, B’s discovery is resolved by sending to B the identification of service A.

Once the consumer has the provider’s identifier, messages are exchanged directly between the provider and the consumer.

A provider can respond with the last sensor value received in the local device or with a dataset between two timestamps. To provide with a dataset, the provider needs to request data to the database service before responding to the consumer. Thus, a consumer-provider request–response can imply a chain of request–response in the choreography system.

#### 3.2.2. Datacentric Pattern

Datacentric subscription is at most one QoS pattern based on asynchronous events. A datacentric service is a bridge between sensors and the service layer. It asynchronously transmits packets of data received from sensors. Filters, error detection, and correction mechanisms can be implemented over datacentric services. Thus, after receiving data from one or several sensors, data are processed by the provider and broadcasted through the choreograph as an asynchronous event. No answer is expected from receivers. Packets are identified by the contained data. Thus, one packet can be received by several consumers. The discovery protocol for such providers responds to the query with the identification mask of the expected packets.

Datacentric patterns are triggered by client subscription. Sending data without an interested receiver only generates noise in the network. Thus, network traffic can be reduced by the selective activation of target services.

**Figure 6:**
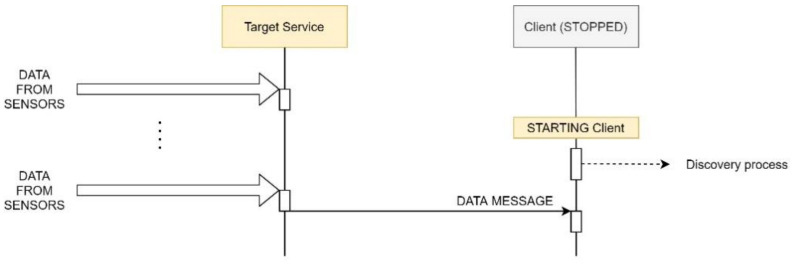
Datacentric pattern: selective activation.

The broker maintains a list of subscribed clients per target service provider. Algorithm 1 describes the ACTIVATION of a target service after the publishing and subscription of a client. In case of a null list, the broker notifies the provider of a NO-ACTIVATION event. Figure 6 depicts the ACTIVATION concept.
**Algorithm 1** Subscribe and unsubscribe updates**Init**: The broker receives a message CASE:  1.Receives a HELLO message from *target_i_*; then *target_i_* list = 0  2.Receives a Subscription to *target_i_*; then *target_i_* list++ and sends an ACTIVATION message  3.Receives an Un-subscription to *target_i_*; then
*target_i_* list--;if list = = 0 then sends a NO-ACTIVATION message

#### 3.2.3. Rule‒Server Pattern

**Figure 7:**
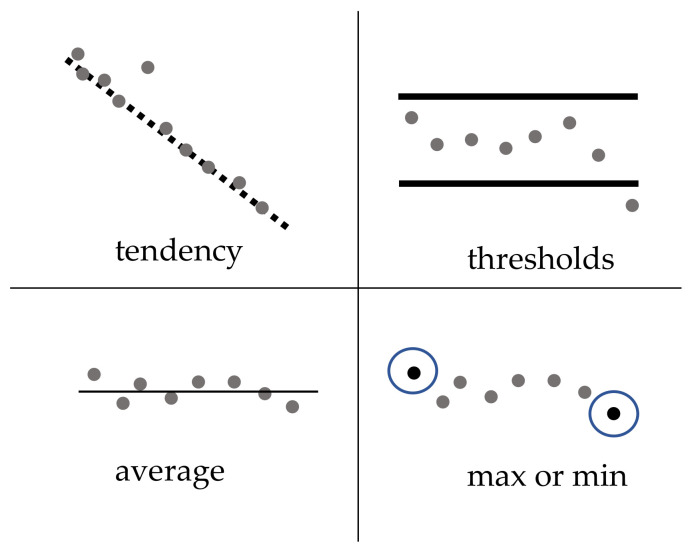
Examples of rules.

The rule‒server pattern consists of a server able to send events when a specific rule matches. It is similar to topic based publish-subscription. Some examples of rules are shown in Figure 7: detection of a value out of tendency, average, or thresholds; detection of the max or min value between two timestamps.

A provider publishes a rule or rules in the broker—for example, a humidity threshold rule lower than *var*%. A rule can involve one or several measurements. Consumers subscribed to the rule specifying *var* value in the delivery process. If the humidity drops below the threshold imposed by the consumer, the provider will send an event message to the consumer service.

The aim of the rule‒server pattern is to reduce network traffic. For instance, when the number of datacentric providers in the system is high, sending a rule assertive packet instead of a continuous delivery loop of data reduces the traffic in the network.

### 3.3. Publishing and Unpublishing

Publishing and unpublishing mechanisms are HELLO and BYE messages, the publishing list, and the lists of subscribed clients per target service provider. Providers and broker interchange information within a request‒response pattern. HELLO and BYE messages are request packets. The expected response is an acknowledge (ACK).

HELLO scope in the body message is assumed to be in natural language with regular expressions. A provider has one or several communication pattern interfaces. Therefore, a new provider that plugs into the system must announce to the broker its interfaces and composition rules if available. The broker updates the publishing list.

In case of a datacentric the provider retains data delivery while there are no subscriptions. As soon as the broker updates the first client subscription to a provider, the broker sends a message to this target service advising the new client.

Similarly, unsubscribe messages update the lists of subscribed clients, causing an asynchronous notification event when the list is empty (Algorithm 1).

BYE messages update the broker’s publishing list. BYE messages are critical for rule‒server clients. While request‒response or datacentric patterns suffer from a delay in detecting the non-reception of data, the rule‒server pattern does not differentiate between a target disconnection and a nonevent state. Therefore, the broker should provide advice to the client about the unavailable provider. The message sent to the client by the broker has the same identification as a provider event message, but the payload string is set to STOP.

## 4. Implementation and Results

The testing system is composed of a set of Raspberry Pi machines, each of which acts as a sink of a LoRa RF-based WSN. The choreograph engine runs in. Net Core and each Raspberry Pi includes a LiteSQL database to store sensor data. Machine interconnection is TCP/IP supported.

The discovery algorithm needs two requests‒response pairs: PROBE and PROBE MATCH, and RESOLVE and RESOLVE MATCH, as shown in Figure 5:

- PROBE comprises the body with the type of sensor measurements, the minimum gathering frequency, and the way in which the consumer prefers to receive data. Message body is described with regular expressions.
- PROBE MATCH nests as many <d: ProbeMatches> sections as found matches, according to the publishing list. Each section includes in the scope the type of measurement, one delivery pattern, and the frequency. Messages are formed of strings, which makes message processing easier for the client. Instances are predefined in the system.
- RESOLVE returns a subcollection of <d: ProbeMatches>. In case of no valid response, the consumer will not start the second pair of requests‒response messages. In case of a rule‒server pattern, the message scope includes the variable *var* that is applicable to the rule. After receiving a RESOLVE package, the broker communicates with the target service provider, reporting the selected rule and variable. This communication between broker and target follows a request‒response pattern. The acknowledge of the target to adopt the rule with the specific variable triggers the RESOLVE MATCH message between broker and client.
- RESOLVE MATCH takes on great relevance in datacentric and request‒response patterns because the broker decides and assigns the provider to balance the load of the different subscribed target services. In the case of rule‒server patterns, RESOLVE MATCH = RESOLVE and the purpose is an acknowledgement.

### 4.1. Data Patterns Analysis

The request‒response pattern is well known and facilitates the connection of new devices and services into the network. However, there is a maximum number of requests that a provider can meet. Table 1 shows a sample of 200 tests and the number of responses per second. The provider responds to a request with a dataset extracted from the local LiteSQL database. Access time to the database increases as the response size increases from 1 to 2M bytes. We observe in Table 1 that service availability is high. However, many system control messages are based on the request‒response pattern, which can increase response latency. On the other hand, datacentric pattern reduces network traffic in case of many consumers requesting the same data. This makes datacentric delivery attractive for networks with high data distribution.

**Figure 8:**
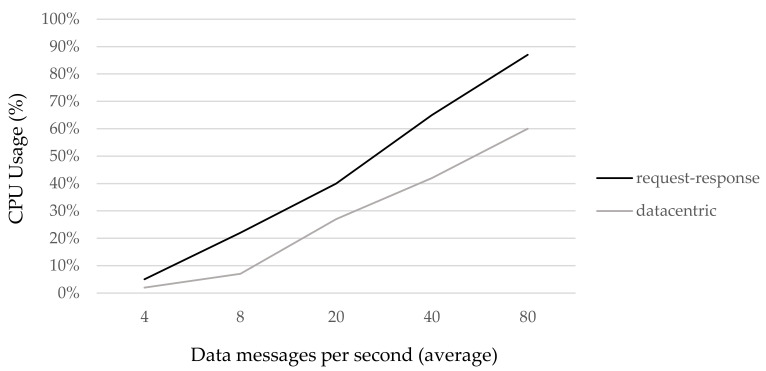
Request‒response vs. datacentric pattern: CPU usage in a Raspberry Pi.

Moreover, while request‒response is a double-message mechanism (one message to request and one message to respond), datacentric and rule‒server are one-message mechanisms, reducing delivery delay and Control Process Unit (CPU) effort. For example, Figure 8 compares the Raspberry Pi CPU usage percentage: request–response intensive versus datacentric intensive to serve the same average number of messages per second.

### 4.2. Latency Interpreter Analysis

The use of regular expressions should also be considered in latency analysis. Interpretation adds complexity to the discovery process.

Strings are composed of predefined simple expressions that describe the type of service. To compare the interpretation latency in this section we define the semantic annotation in Table 2 for the discovery scope: *serv* = *service*, *p* = *pattern*, *f* = *frequency*, *ch* = *channel*, *r* = *rule*, *m* = *model*.

For example, a rule‒server provider that serves humidity data according to a certain threshold, which is received from a sensor every 10 min (sensor delivery frequency), will compose a body HELLO message with strings as follows:

<d:Types> i: humidity </d:Types>

<d:Scopes> serv:///p = RULESERV, f = 10m, r = thrs </d:Scopes>

**Figure 9:**
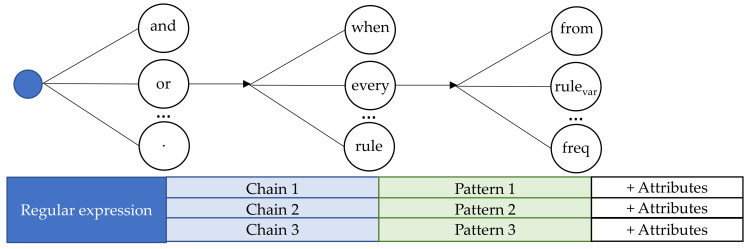
Regular expressions interpreter: example.

A provider could offer more than one delivery pattern, or a consumer could accept more than one pattern. In such a case, the use of strings requires nested descriptions of the message body. Each pattern is described separately. The use of regular expressions allows integrating new services with a minimum semantics knowledge of atomic words. For example, in Figure 9 we developed a testing interpreter of regular expressions in three stages. The regular expression is the input in the interpretation process. In each phase of Figure 9, the interpreter uses a list of atomic words or expressions. Expressions such as “and,” “or,” or punctuation marks divide the input chain into several chains, each of which is a unique potential delivery pattern. A unique pattern needs to be identified for each chain. Each pattern is associated with a list of words. Expressions such as “when,” “every,” or “rule” are examples of words that refer to the pattern type. Each pattern has a list of associated words. The words contained in each list are exclusive. It means that a word can only be contained in one list. If the process finds two words of the same chain in different lists, it fails.

Finally, expressions such as “from,” “tendency,” “threshold,” “maximum,” “minimum,” and “frequency”, among others, are pattern attributes. There are exclusive attributes assignable only to a pattern and attributes that can be found in two patterns. In the first case, attributes also serve to identify the pattern.

Therefore, the process is based on the completeness of the interpretation lists. In future work, the interpreter could allow more complexity in the parsed expressions. However, the more complex, the more delay it will introduce into the system. We will use the one in Figure 9 for comparison.

The simplest HELLO message described with a regular expression is a request‒response. For example, a blank scope will be interpreted to provide the most recent data, while “dataset” in the scope will be interpreted to provide the dataset between two timestamps:

1. XFIPAMSG_payload {
2.  <d:Hello>
3.   <a:EndpointReference>
4.     <a:Address>Service.address</a:Address>
5.   </a:EndpointReference>
6.   <d:Types>i: humidity </d:Types>
7.   <d:Scopes> dataset </d:Scopes>
8.   <d:MetadataVersion>0000</d:MetadataVersion>
9.  </d:Hello>
10. }

Patterns such as datacentric or rule‒server include more information. Moreover, a provider published out of the local network includes complementary XAddrs parameters:

1. XFIPAMSG_payload {
2.  <d:Hello>
3.   <a:EndpointReference>
4.     <a:Address> Service.address </a:Address>
5.   </a:EndpointReference>
6.   <d:Types> i:humidity </d:Types>
7.   <d:Scopes>
8. Rule THRESHOLD percentage
9. </d:Scopes>
10.   [<d:XAddrs> xs:localhost:9020 xs:192.168.1.215:9031 </d:XAddrs>]
11.   <d:MetadataVersion> 0001 </d:MetadataVersion>
12. </d:Hello>
13. }

**Figure 10:**
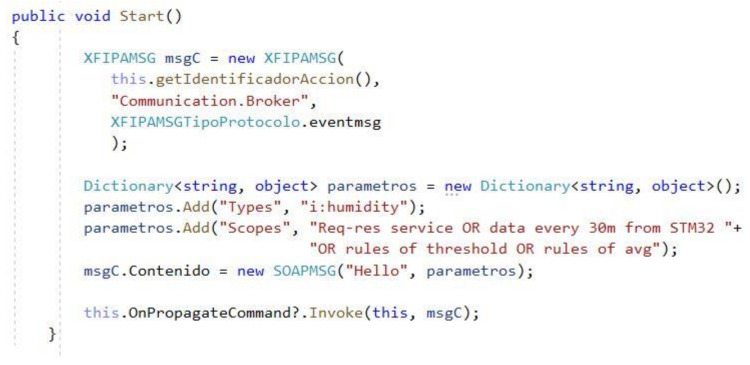
Message built with a regular expression in the scope: C# example.

Compared to messages generated with strings of basic expression, regular expressions do not need to nest sections within the body of the message as many options are offered. The scope contains the overall information. For example, the following client probe message sets four chains inside the scope: <d:Scopes> Req-res service OR data every 30m from STM32 OR rules of threshold OR rules of avg</d:Scopes>. Message composition in shown in Figure 10:

Message size with a regular expression is 209 bytes. The size of the same message but with four nested PROBE requests is 494 bytes. The difference is significant in complex descriptions which implies an advantage.

On the other hand, Table 3 compares several HELLO messages. RE is the broker delay in resolving a message written with a Regular Expression. S is the broker delay with strings in semantic annotation. The table shows both the scope written with regular expressions and the equivalent composition with strings.

Similarly, PROBE messages to the broker are built with regular expressions. Table 4 compares the broker delays to different client PROBES, depending on the scope being a regular expression or using strings.

As shown in Table 3 and Table 4, although the use of regular expressions opens a great opportunity in the building of blind hot plugging, the delay in the interpretation of these messages is high compared with the use of typical message scopes.

### 4.3. CPU Usage and Memory Analysis

**Figure 11:**
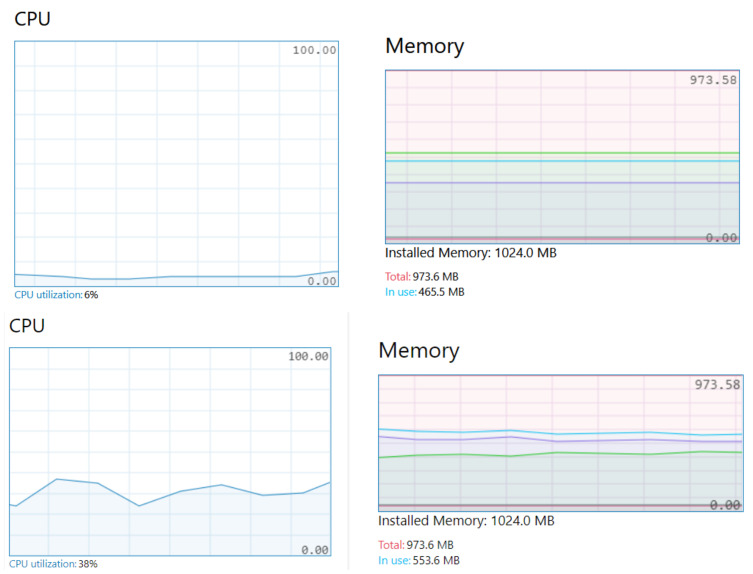

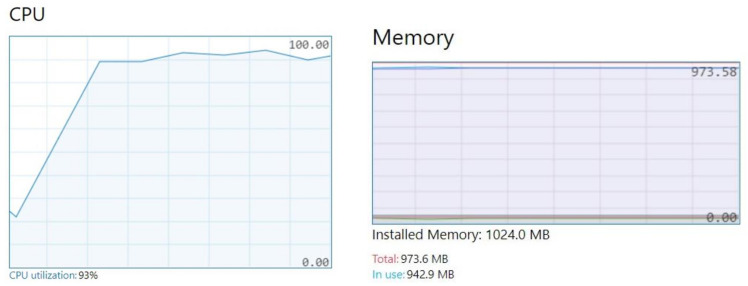
CPU usage and memory consumption under test.

Finally, we analyzed the impact of the choreograph engine and the discovery service on CPU usage and memory. We deployed two Raspberry Pi machines: RP1 and RP2. The analysis included four steps to stress RP1 machine and analyze the maximum load it can hold:

(1) Figure 11 (upper) shows a machine hosting eight microservices but without data exchange. Services require half of the Raspberry Pi memory.
(2) Figure 11 (middle) shows the eight microservices exchanging data. Three services are request–response data consumers; one service is request–response data provider; four services are datacentric providers. Data exchange is continuous, and messages are produced in a ratio between 0.4 and 1.0 s. The CPU usage increases up to 38%.
(3) Figure 11 (lower) includes a ninth microservice, the broker. The experiment stresses CPU and memory. The broker receives on average 10 discovery probes per second from services hosted in RP2. CPU raises up to a 95% and memory usage near 100%. We observe that, in addition to discovery process times shown in Table 4, internal choreography message passing introduces a latency λ of 29.4 ms average per petition. The system is stable but λ ranges from 2 to 59 ms with a σ standard deviation of 18.07 ms in 135 samples.
(4) Finally, data exchange between consumers and providers is stopped. Input and output broker messages maintain CPU activity up to 95%. The experiment reduces λ to 4.97 ms and σ to 2.52 ms in 250 samples.

Therefore, the impact of the choreography messaging layer is variable and λ depends on the overall activity of the machine. This can be a drawback in real-time systems design, although clustering is also possible to limit the number of services per cluster.

## 5. Discussion

Intermediate layers in the IoT architecture, the edge and fog layers, receive a large amount of information and act as an intercommunication gateway between things and cloud systems. The edge layer is composed of both single IoT devices and edge machines or gateways able to execute different microservices in the same machine. Such machines can improve systems performance by processing and filtering data to reduce traffic to the cloud and carrying out automatic decisions, among others. However, the growth in number of IoT devices implies the growth in complexity of edge machines. In this paper, choreography is explored as a well-known service composition model in large computational services, to support complex distributed edge systems.

Under choreography message exchange models, services are location agnostic of other services. Data exchange is abstracted from the transport layer. Thus, it allows the flexible relocation of services among the cyber–physical components, as well as the incorporation of new services without reprogramming the existing ones. To explore this advantage at the edge layer, we should face different issues.

On the one hand, IoT systems present a high heterogeneity in terms of data demand. Some data are scarcely required, while others are in high demand. Therefore, the same data exchange pattern between data providers and consumers cannot always be applied. We need mechanisms that allow to choose or discover dynamically the subscription pattern type of a service consumer according to the existing providers but reducing the number of requests‒responses in favor of datacentric and rule‒server patterns.

This paper discusses the request‒response pattern, proposing as alternatives (but not wholesale replacements) other models: datacentric and rule‒server. Both models are focused on IoT systems with high demand for distributed data streams among edge machines or gateways. For example, a datacentric pattern combined with the use of reception masks allow for the sending of one-to-many data. Moreover, we observe that CPU usage also decreases with datacentric intensive use.

On the other hand, discovery mechanisms are necessary to advance in the design and improvement of flexible edge layers. We propose an implementation based on a global vision of the system and centralized in a specific broker service. As an advantage, the broker can be supplied with interpretation features to associate provider–consumer to balance the load of the services and reduce traffic. However, we have seen that the centralization in a unique machine limits the scalability of the system. The delivery latency is conditioned by the volume of packages that moves the choreography engine. Thus, to support scalability it will be necessary to explore in the future decentralization mechanisms.

Using regular expressions reduces the size of the messages but we observe that it generates a non-negligible CPU overload. It is an open issue, and a solution could be the use of dedicated machines on clustering. For example, web service discovery current trends in clustering [54,55] are focused on effectively narrowing down the searching scope. Clustering techniques could be applied to decentralize the discovery service and to avoid single point failures in future.

## 6. Conclusions

There are few works that provide solutions and evaluate the use of choreography models on low-cost machines with limited features such as the Raspberry Pi. In this paper, these machines were tested to support a data exchange engine that allows collaborative dataflows of choreography models.

The paper presented an adaptation of the typical patterns of data exchange in IoT, considering the two most frequent: request–response and datacentric. In addition, event services triggered by rules based on sensor input data were also considered.

These three patterns agreed on a mechanism for the discovery of services or microservices providers of data and datasets. The WS-Discovery managed discovery rules were adapted to reduce the burden on the service broker by limiting it to provider–consumer matching, while the dataflow is resolved by the choreography engine and its exchange rules.

To reduce the size of discovery probe and hello messages, the use of regular expressions is built into an interpreter method. In this paper, the CPU and memory resources required to support this facility were analyzed, as well as the difference in latency interpretation compared to the traditional string-based messages. The CPU effort depends on the number of discovery requests per second. We stressed the CPU with about 10 requests per second and introduced an additional latency in the interpreter response of 29.4 ms. Both the latency and the number of requests served per second can be improved if the broker is hosted on the Raspberry Pi without sharing CPU or memory with other service providers–consumers.

Therefore, there are open issues such as reducing CPU consumption of the interpreter or such as the distribution of the discovery effort in clusters or similar.

In general, this paper is novel because of the distributed functionality and collaborative data model, the adaptation of the typical messaging mechanisms in IoT, and the use of mechanisms to exploit the possibilities of hot blinded scalability at the IoT edge layer.

## Figures and Tables

**Table 1:** Request‒response pattern: messages replied to per second.

Response Payload Size	Number of Responses	DB Access_Max Time
1 byte	82	5 ms
10 bytes	80	5 ms
100 bytes	75	5 ms
1K bytes	42	20 ms
10k bytes	38	20 ms
100k bytes	20	50 ms
1M bytes	16	50 ms
2M bytes	7	100 ms

**Table 2:** Semantic annotation used in the testing examples.

Annotation	Description	Domain	Value
p	Pattern: request–response, datacentric and rule–server	String	REQRES, DATACENTRIC, RULESERV
f	Time: milliseconds, seconds, minutes, hour	String	ms, s, m, h
ch	Data ID (HELLO messages)	Integer	[00...99]
r	treshold, average, tendency, maxmin	String	thrs, avg, tnd, max, min
m	sensor model	String	Manufacturer ID

**Table 3:** RE and S latency comparison: HELLO messages.

RE Scope	S Scopes	RE	S
Send data on demand	p = REQRES	24 ms	7 ms
Request type service frequency 5 m	p = REQRES + f = 5 m	19 ms	8 ms
Req‒resp service	p = REQRES	24 ms	9 ms
Hello, I am a req service	p = REQRES	21 ms	10 ms
Send data every 30 m from DHT11 on channel 00	p = DATACENTRIC + m = DHT11 + f = 30 m + ch = 00	50 ms	27 ms
Send every 1 h from SGP40 on ch 1	p = DATACENTRIC + m = SGP40 + f = 1 h + ch = 01	84 ms	32 ms
Data frequency 30 m on channel 02	p = DATACENTRIC + f = 30 m + ch = 02	64 ms	19 ms
Accept rules of threshold	p = RULESERV + r = thrs	60 ms	17 ms
Accept rules of average	p = RULESERV + r = avg	62 ms	21 ms
Req‒resp service and accept tendency rules	String_1: p = REQRES String_2: p = RULESERV + r = tnd	113 ms	78 ms
Average rules, data every 30 m on ch 04	String_1: p = DATACENTRIC + f = 30 m + ch = 04 String_2: p = RULESERV + r = avg	135 ms	89 ms

ch: channel; thrs: threshold.

**Table 4:** RE and S latency comparison: PROBE messages.

1	PROBE S Scopes	PROBE MATCH * Coherent with TYPE Field	RE	S
-	-	REQRES service	4 ms	3 ms
Request every 1 h	p = DATACENTRIC + f = 1 h	2 DATACENTRIC services	17 ms	9 ms
Data every 1 h from SGP40	p = DATACENTRIC + f = 1 h + m = SGP40	1 DATACENTRIC service	20 ms	10 ms
Data from SGP40	String_1: p = DATACENTRIC + m = SGP40 String_2: p = REQRES + m = SGP40	1 DATACENTRIC service	27 ms	21 ms
Threshold service max 30 and min 14	p = RULESERV + r = avg + arg = 30, 14	1 RULESERV service	128 ms	22 ms
Avg service between *A* and *B*	p = RULESERV + r = avg + arg = *A*, *B*	2 RULESERV services	142 ms	69 ms
Data every 30 m and a thrs rule with max 31 and min 23	String_1: p = DATACENTRIC + f = 30 m String_2: p = RULESERV + f = 30 m + r = thrs + arg = 31, 23	1 DATACENTRIC service + 1 RULESERV service	167 ms	96 ms

* PROBE MATCH means the number of services found in our system that meet the probe request. arg: arguments; thrs: threshold; avg: average.

## Data Availability

The data contained in the paper are available from the authors.
